# Direct dioxygen evolution in collisions of carbon dioxide with surfaces

**DOI:** 10.1038/s41467-019-10342-6

**Published:** 2019-05-24

**Authors:** Yunxi Yao, Philip Shushkov, Thomas F. Miller, Konstantinos P. Giapis

**Affiliations:** Division of Chemistry and Chemical Engineering, California Institute of Technology, Pasadena, CA 91125 USA

**Keywords:** Chemical physics

## Abstract

The intramolecular conversion of CO_2_ to molecular oxygen is an exotic reaction, rarely observed even with extreme optical or electronic excitation means. Here we show that this reaction occurs readily when CO_2_ ions scatter from solid surfaces in a two-step sequential collision process at hyperthermal incidence energies. The produced O_2_ is preferentially ionized by charge transfer from the surface over the predominant atomic oxygen product, leading to direct detection of both O_2_^+^ and O_2_^−^. First-principles simulations of the collisional dynamics reveal that O_2_ production proceeds via strongly-bent CO_2_ configurations, without visiting other intermediates. Bent CO_2_ provides dynamic access to the symmetric dissociation of CO_2_ to C+O_2_ with a calculated yield of 1 to 2% depending on molecular orientation. This unexpected collision-induced transformation of individual CO_2_ molecules provides an accessible pathway for generating O_2_ in astrophysical environments and may inspire plasma-driven electro- and photo-catalytic strategies for terrestrial CO_2_ reduction.

## Abstract (web summary)

Carbon dioxide can dissociate via different pathways depending on the amount of available energy. Here the authors investigate carbon dioxide collisions with metal substrates and observe a pathway producing molecular oxygen, which might explain the presence of oxygen in abiotic environments.

## Introduction

Although plentiful in modern Earth’s atmosphere, molecular oxygen is extremely rare in space. Only trace amounts have been found elsewhere in our solar system^1–3^ and in interstellar clouds^4,5^. The recent discovery of abundant O_2_ in the coma of comet 67P/CG^6^ has rekindled interest in abiotic reactions, occurring in extreme environments, which release O_2_ from compounds, such as H_2_O, CO_2_, CO, silicates, and metal oxides. Such reactions may offer competing explanations for the origin of O_2_ in comets, in the upper atmosphere of Mars, and in Earth’s prebiotic atmosphere^7–9^. They may also present alternative ways for resource utilization related to space travel, such as generation of O_2_ from CO_2_ for making Mars habitable. Finally, new strategies for CO_2_ activation may be inspired by such reactions.

The dissociation of CO_2_ proceeds via multiple pathways depending on available energy. The partial dissociation reaction, CO_2_ → CO + O (^3^P or ^1^D), has the lowest energy requirement (5.43 or 7.56 eV)^10^; it has been extensively studied in photochemistry and in heterogeneous catalysis under thermal activation conditions^11,12^. Full dissociation to C + O + O involves the cleavage of both C–O bonds and requires 16.46 eV. Other pathways may be possible at intermediate energies, such as the exotic reaction: CO_2_ → C(^3^P) + O_2_(^1^Σ_g_), which entails extensive intramolecular rearrangement of the CO_2_ molecule. Calculations have suggested that this reaction proceeds on the ground-state potential energy surface, by first forming a cyclic CO_2_ intermediate [c-CO_2_(^1^A_1_)], which then rearranges into a collinear COO(^1^Σ^+^) intermediate on its way to dissociation into C + O_2_^10^. The first step in this channel involves bending of the CO_2_ molecule to bring the two O atoms in close proximity, which requires close to 6 eV of internal energy^13^.

Although inaccessible by thermal activation, transitions to electronically excited and anionic states of CO_2_ can bend the molecule as a first step to O_2_ production. Indeed, pioneering experiments employing VUV photo-excitation^14–16^ and electron attachment^17,18^ have shown that dissociation of CO_2_ into C(^3^P) + O_2_(X^3^Σ_g_^−^) is possible, as evidenced by the detection of the complementary atomic C^+^ or C^−^ fragment. Further confirmation of the exotic pathway, however, remained elusive as neutral or ionized O_2_ products were not detected. Using ion-beam scattering methods and numerical simulation techniques, we demonstrate here a different way to drive the direct reduction of CO_2_ to O_2_ with in situ detection of ionized O_2_ products. The process involves a previously unknown intramolecular reaction pathway, which occurs in energetic CO_2_ ion–surface collisions with a surprising lack of dependence on either the nature of the surface or the surface temperature. As such, the reaction may be relevant for astrophysical environments, such as comets, moons, and planets with CO_2_ atmospheres.

## Results

### Carbon dioxide scattering experiments and kinematic analysis

**Fig. 1:**
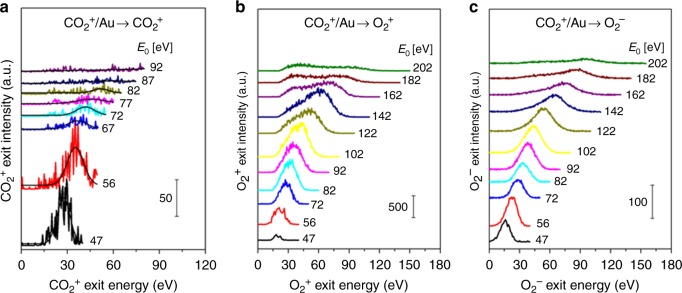
Dynamic production of O_2_^±^ in CO_2_^+^ collisions on Au. Scattered product kinetic energy distributions of **a** CO_2_^+^, **b** O_2_^+^, and **c** O_2_^−^ ion exits from CO_2_^+^/Au for various CO_2_^+^ beam energies (*E*_0_) as annotated on each panel. Signal intensities in **b** and **c** cannot be compared to each other due to differences in detector bias

We first demonstrate the formation of O_2_ in hyperthermal CO_2_^+^/Au collisions by plotting kinetic energy distributions of three scattered molecular ion products: CO_2_^+^, O_2_^+^, and O_2_^−^ for various CO_2_^+^ incidence energies (*E*_0_). Very weak signal of scattered CO_2_^+^ is detected for *E*_0_ < 80 eV (Fig. 1a). The CO_2_^+^ exit energy peak varies in proportion to *E*_0_, thus implying a ballistic or impulsive rebound from the surface and thereby precluding physical sputtering as its origin. Observation of this “dynamic” CO_2_^+^ signal is important, not only for proving that some CO_2_ survives the surface encounter but also for unraveling the collision sequence of the constituent atoms. Strong signal of scattered O_2_ ions is also observed (Fig.1b, c). The O_2_^+^ and O_2_^−^ exit energies represent a large fraction of the incidence energy (57%) and increase monotonically with *E*_0_ over a larger range than scattered CO_2_^+^. The O_2_ ion signal intensity exhibits a maximum at *E*_0_ ~ 100 eV. Above that, only the O_2_^+^ distribution develops a shoulder (i.e., exit at ~30 eV) from physical sputtering.

The detection of fast O_2_ ion products is surprising. Neither sputtering of surface O_2_ nor O-atom abstraction reactions (Eley–Rideal) can explain their formation, because both mechanisms would produce O_2_ at much lower exit energies (see the section “Methods”). A remaining possibility to be explored here is dynamic formation of O_2_ through dissociation of CO_2_. Dynamic partial and full dissociation of CO_2_ is in fact consistent with the other detected products, including CO^+^, CO^−^, O^+^, O^−^, and C^+^ (Supplementary Fig. 1). The exit energy of the CO^+^, CO^−^, and O^−^ fragments varies linearly with incidence energy, consistent with dynamic formation during the surface collision. In contrast, the O^+^ and C^+^ peaks show little dependence on *E*_0_, suggesting a different origin, i.e., sputtering^19^. Scattered C^+^ products appear at *E*_0_ > 80 eV, confirming full dissociation.

**Fig. 2:**
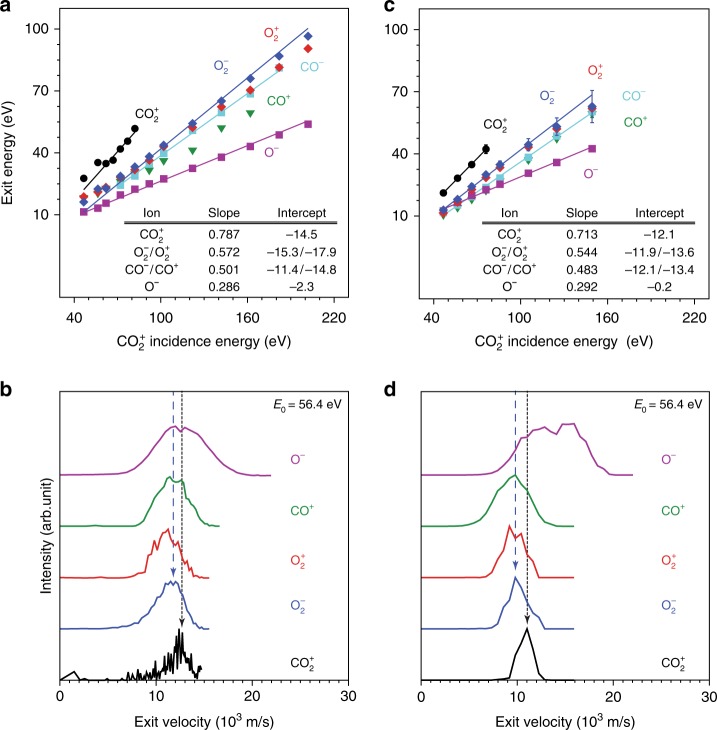
Kinematics and velocity analysis of CO_2_^+^ scattering on Au. **a** Experimental exit energies of CO_2_^+^, O_2_^+^, O_2_^−^, CO^+^, CO^−^, and O^−^ ions from CO_2_/Au collisions as a function of CO_2_^+^ incidence energy. All points represent the peak of the respective energy distribution obtained from Gaussian fitting of the experimental data. All solid lines represent one-parameter linear fittings with BCT-derived slopes. No fittings are shown for O_2_^+^ and CO^+^ data because of overlap with their negative ion counterparts. **b** Experimental velocity distributions of select scattered product ions for *E*_0_ = 56.4 ± 2.5 eV. **c** Calculated exit energies of CO_2_^+^, O_2_^+^, O_2_^−^, CO^+^, CO^−^, and O^−^ ions from MD simulations of CO_2_/Au collisions as a function of CO_2_^+^ incidence energy; slopes and intercepts listed in the inset are two-parameter best-fittings. The error bars represent one standard deviation across 10 samples of 2000 trajectories each from the ensemble of molecular dynamics trajectories. **d** Calculated velocity distributions from MD simulations for select scattered ion products at *E*_0_ = 56.4 eV. Vertical dashed lines in (**b**) and (**d**) indicate alignment with respect to the CO_2_^+^ (dashed black line) and O_2_^−^ (dashed blue line) peaks

The presence of dynamically exiting CO_2_^+^ ions enables use of kinematics^20^ to clarify the scattering mechanism. Binary collision theory (BCT) allows calculation of the kinematic factor, defined as the fraction of incident energy retained by a scattered product exiting the surface. In the simplest possible model, CO_2_^+^ scatters as a whole molecule, i.e., a hard sphere with atomic mass of 44 Da. Under this assumption, BCT predicts a kinematic factor of 0.6349, which fits the data poorly (Fig. 2a) as may be expected given the quasi-linear nature of the triatomic CO_2_^+^ ion^21,22^. We consider next a kinematic model in which—as for diatomic molecules scattering on metal surfaces^23^—the leading O atom first collides with a surface Au atom, followed by a second collision of the CO moiety without prompt dissociation of the CO_2_ molecule. Applying BCT to this sequential-collision model yields a kinematic factor of 0.7870, which agrees very well with the CO_2_^+^ exit energy data (Fig. 2a, black line).

The average exit energies for all remaining scattered products are also plotted in Fig. 2a. Potential origins for such species include partial or full dissociation of CO_2_ and surface sputtering of adsorbed CO_2_ fragments. While some sputtering is indeed observed at high *E*_0_ (>140 eV), kinematic analysis of the exit energy data provides strong evidence for impulsive dissociation of the CO_2_ molecule^24^. Assuming delayed fragmentation of the CO_2_ parent^24^, the kinematic factors of the CO, O, and (possibly) O_2_ daughter products can be calculated from energy conservation to be 0.5724, 0.5008, and 0.2862, respectively. These factors are used as fixed slopes in one-parameter fittings of the respective data points (adjustable intercept). We find that the calculated slopes fit the O_2_^±^, CO^±^, and O^−^ ion exit data very well (Fig. 2a lines), indicating that the latter ions are all dissociation products of CO_2_. On the contrary, the O^+^ and C^+^ data are not linear with respect to *E*_0_, suggesting formation by other processes.

Velocity analysis for the observed scattered species provides further evidence regarding the collision mechanism. Figure 2b compares the ion distributions of various peaks for *E*_0_ = 56.4 eV. The exit velocities of scattered CO^+^, O_2_^+^, O_2_^−^, and the slower part of the O^−^ distributions overlap, suggesting a common origin. However, the O^−^ distribution is noticeably broader, extending to higher exit velocities, which suggests alternative formation channels. The O_2_ ion products exit with velocities lower than CO_2_^+^ owing to inelasticity from breaking of chemical bonds and non-resonant surface ionization.

Although the kinematic analysis indicates conclusively that some CO_2_ scatters intact after a two-step sequential collision of the O and CO moieties, it leaves various aspects of the O_2_ formation mechanism unresolved. In particular, since the experiment is limited to observing ions, we are unable to assess how much neutral O_2_ is produced. Moreover, the kinematic analysis cannot shed light on whether O_2_ is formed via an electronically adiabatic or non-adiabatic mechanism, nor can it disentangle the collision-induced pathways that underlie the exit velocity distributions of the ionic fragments. To address these questions, we next turn to first-principles molecular dynamics (MD) simulations.

### MD simulations of carbon dioxide collisions with gold

MD trajectories for the scattering of CO_2_ on Au(111) are performed in the experimental scattering geometry, with CO_2_ evolving on the ground singlet potential energy surface under the assumption that incoming CO_2_^+^ ions are neutralized before the hard collision. Facile neutralization occurs via resonant electron tunneling^24–26^ from the metal surface to the molecular cation because the molecular level of CO_2_ (−13.8 eV) lies well within the occupied band of Au (−5.3 to −15.3 eV). Electron transfer from and to the surface is explicitly included in the simulations to also account for ionization of neutral collision products. The calculated exit energies of the products are plotted in Fig. 2c along with linear two-parameter fits. The slopes obtained from this fitting procedure compare very well to those determined from BCT (Fig. 2a). For example, the exiting CO_2_^+^ has a calculated slope of 0.713 vs. the experimental value of 0.787. Negligible CO_2_ is found to survive for *E*_0_ > 80 eV, consistent with the lack of experimental signal at these energies. All other calculated slopes agree well with the experimental values; for instance, compare the slope of 0.54 ± 0.02 vs. the experimental value of 0.57 for the O_2_^−^ line. These results indicate broad agreement between the simulations and the scattering kinematics.

The formation of ions detected in the experiment requires surface ionization, which influences the yields of the ionic products. The MD simulations demonstrate a substantial enrichment of O_2_^−^ ions over O^−^, resulting from the exponential dependence of the ionization probability on the coupling to the metal surface (Supplementary Fig. 2, red curve), which can reach ~30%, comparable to the experimentally derived yield of 33% (Supplementary Fig. 2, blue curve).

The agreement between experiment and simulations is further demonstrated by comparing the ion exit velocity distributions at *E*_0_ = 56.4 eV (Fig. 2d). Although the experimental peak positions appear systematically at somewhat larger velocities than the calculated ones, the distributions agree very well with respect to relative position of the peaks. In particular, both simulations and experiment find the CO^+^ and O^−^ velocity distributions to be broadened, both find the O_2_^+^ and O_2_^−^ distributions to be similar with the cation exiting slower than the anion, and both find CO_2_^+^ to exit with higher velocity than the ionized O_2_ products. The agreement suggests that the simulations provide a strong foundation for analyzing the reaction mechanism of the direct CO_2_ conversion to O_2_.

**Fig. 3:**
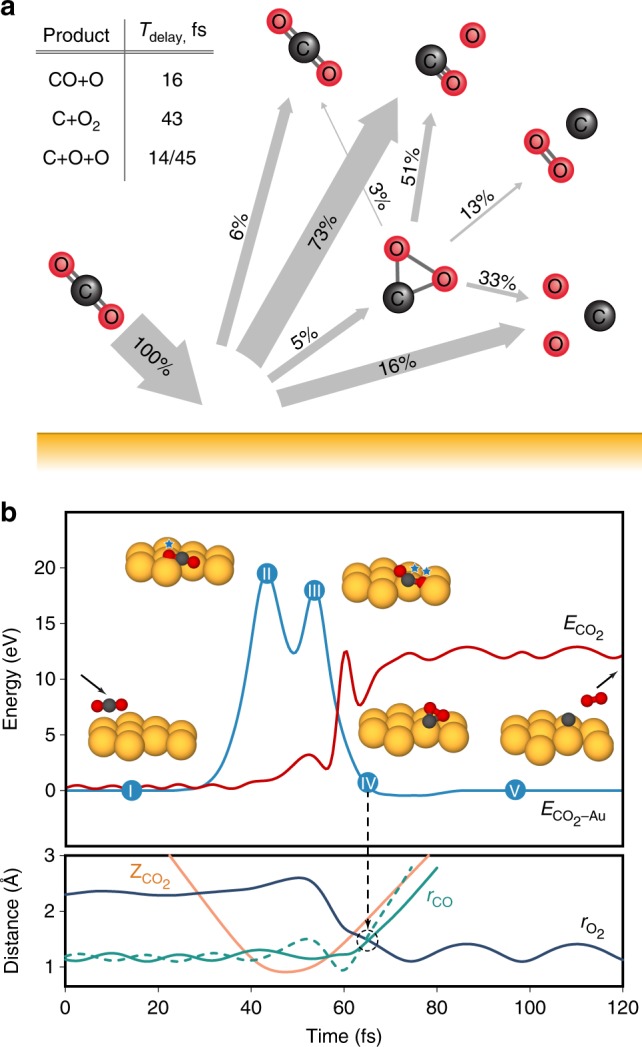
Product yields and energetics of CO_2_ scattering on Au. **a** Calculated yields of neutral dissociation products from a statistical analysis of an ensemble of CO_2_ scattering trajectories on Au(111) at *E*_0_ = 56.4 eV. Inset: average delay times (*T*_delay_) of the dissociation channels relative to the point of closest approach of CO_2_ to the surface. **b** Energetics along an illustrative CO_2_ collision trajectory, which leads to O_2_ formation on Au at *E*_0_ = 56.4 eV. Curve identification: CO_2_–Au interaction energy (light blue), CO_2_ potential energy (red), CO_2_ center-of-mass distance from the metal surface (orange), O–O bond length (dark blue), and C–O bond lengths (full and dashed green lines). Insets I–V: Select configurations of the CO_2_ surface geometry along the scattering trajectory

An ensemble of 20,000 CO_2_-on-Au collision trajectories were performed for each incidence energy, resulting in a variety of dissociation products, including O_2_ (Fig. 3a). Prior to the mechanistic ensemble analysis, it is instructive to review one representative trajectory that leads to collisional O_2_ formation (Fig. 3b). Select configurations are shown as insets, along with the CO_2__–Au_ interaction energy, ${E}_{{\mathrm{CO}}_{2}}$_–Au_, and the CO_2_ internal energy, ${E}_{{\mathrm{CO}}_{2}}$, as a function of time. The incoming CO_2_ molecule is vibrationally excited (inset I). As the center-of-mass distance to the surface, ${Z}_{{\mathrm{CO}}_{2}}$, decreases, the molecule penetrates the repulsive potential wall of the surface and ${E}_{{\mathrm{CO}}_{2}}$_–Au_ increases steeply. During this encounter, one of the O atoms of CO_2_ strikes a surface Au atom, giving rise to the first peak in the ${E}_{{\mathrm{CO}}_{2}}$_–Au_ curve (inset II). This collision occurs before ${Z}_{{\mathrm{CO}}_{2}}$ reaches a minimum at the apsis point. As the O atom rebounds, the CO moiety collides with a different Au atom, causing a second peak in the ${E}_{{\mathrm{CO}}_{2}}$_–Au_ curve (inset III), which occurs after the apsis. As a result of the impulsive energy transfer during the collision, the rebounding CO_2_ undergoes substantial intramolecular rearrangement portrayed by the bond distance evolution in Fig. 3b. The O–O distance, ${r}_{{\mathrm{O}}_{2}}$, decreases while the C–O distances, *r*_CO_, simultaneously increase, reaching a point along the trajectory where CO_2_ acquires a triangular configuration with nearly equal bond lengths (vertical dashed line). This strongly bent CO_2_ intermediate (inset IV) has a significant amount of internal energy, ${E}_{{\mathrm{CO}}_{2}}$, and promptly dissociates to give a free C atom and a vibrationally hot O_2_ molecule (inset V). The complete CO_2_ collision trajectory discussed in Fig. 3b can be viewed in the Supplementary Video. The formation of O_2_ depicted by this representative trajectory proceeds by delayed fragmentation following the two-step sequential collision of CO_2_ with the surface. This mechanism is consistent with the assumptions of the kinematic model used earlier to explain the experimental data in Fig. 2a, b.

**Fig. 4:**
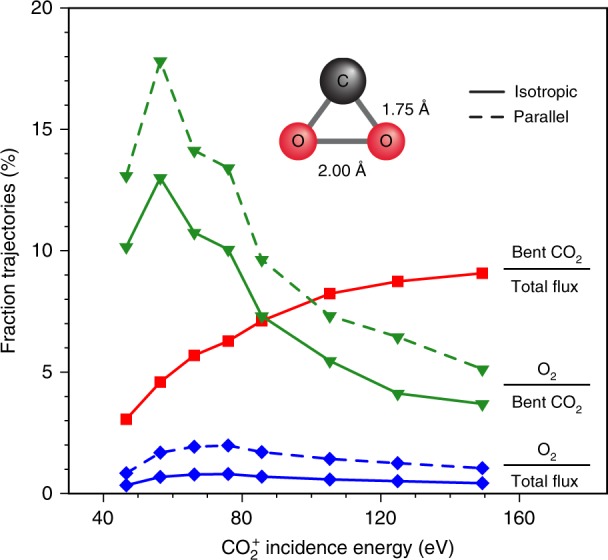
Reaction probabilities for symmetric dissociation of CO_2_ on Au. Calculated total O_2_ yields for isotropic (solid blue line) and parallel (dashed blue line) orientation of incident CO_2_ as a function of incidence energy. Also shown: Fraction of all trajectories reaching the strongly bent CO_2_ intermediate state (solid red line); and fraction of the latter trajectories, which dissociate symmetrically to C + O_2_ for isotropic (solid green line) and parallel (dashed green line) orientations. Inset: average geometry of the intermediate state computed from the lowest energy, bent CO_2_ configurations visited by the scattering trajectories

The calculated reaction yields of the various collision-induced dissociation channels of CO_2_ at *E*_0_ = 56.4 eV are shown in Fig. 3a. As expected for this low incidence energy, the partial dissociation channel dominates the reaction yield with 73% of all MD trajectories taking that pathway. The complete dissociation channel is second at 16%. A small fraction of the incoming CO_2_ (6%) survives the collision in correspondence with experimental detection. Approximately 5% of all trajectories lead to the strongly bent intermediate state—the precursor to O_2_ formation—which is characterized by C–O and O–O bond orders exceeding 0.7. This intermediate state fragments primarily via partial dissociation (51%) followed again by complete dissociation, albeit now with a higher yield (33%). Remarkably, one in eight (13%) of the strongly bent CO_2_ molecules produces O_2_. The overall neutral yield of the symmetric dissociation channel, CO_2_ → C + O_2_, amounts to 0.6% at *E*_0_ = 56.4 eV. Upon increasing incidence energy, the neutral O_2_ yield obtained from the ensemble of isotropically oriented incident CO_2_ molecules reaches 0.8 ± 0.2% for *E*_0_ ~ 70 ± 15 eV (Fig. 4, blue line). Also it is clear from the figure that the fraction of O_2_-producing trajectories increases substantially once the strongly bent CO_2_ intermediate state is reached (Fig. 4, green line) and this fraction peaks at around 13% for *E*_0_ ~ 55 ± 10 eV. The smaller total neutral O_2_ yield results from the small fraction of linear CO_2_ molecules reaching the strongly bent state (Fig. 4, red line). By preferentially orienting incoming CO_2_ molecules (axis parallel to the surface), the fraction of O_2_-producing trajectories increases to ~2% (Fig. 4, dashed blue line) resulting from an increase of the dissociation probability of the strongly bent state to O_2_ (Fig. 4, dashed green line). These findings suggest that activation of bending and symmetric stretching motion of CO_2_ prior to the collision may facilitate both the population of the strongly bent state and its dissociation to O_2_ leading to a significant increase in the total neutral O_2_ yield.

The timescales for bond breaking and formation in the collision-induced dissociation reactions were evaluated for *E*_0_ = 56.4 eV and are reported in the inset of Fig. 3a. The average delay times reveal that both partial dissociation and the first C–O bond-breaking event in complete dissociation occur promptly after the apsis. In contrast, the formation of the strongly bent CO_2_ intermediate state and its fragmentation to O_2_ occur on a longer timescale, to allow for the significant intramolecular rearrangement that precedes symmetric dissociation. This is again consistent with the assumption of delayed fragmentation used in the kinematic modeling. The second C–O bond breaking in the complete dissociation channel is also delayed, irrespective of the degree of bending of CO_2_. The different timescales of the collisional reactions explain the widths of the observed exit velocity distributions. For instance, O atoms produced in prompt partial and delayed complete dissociation, have different velocity profiles, giving rise to a considerably broader O^−^ velocity distribution (Fig. 2d). In particular, prompt partial dissociation involves direct scattering of O atoms from the much heavier Au target, producing faster O-atom exits owing to inefficient momentum transfer. On the other hand, the second C–O bond breaking involves dissociation of the more massive, recoiling CO moiety of CO_2_, which gives off slower O atoms (Supplementary Fig. 3). Moreover, the narrow velocity profiles of the molecular O_2_ ions stem from CO_2_ scattering as a whole molecule, which breaks apart unimolecularly during the rebound from the surface.

## Discussion

The convergent analysis and agreement among experiment, kinematics, and first-principles MD simulations presented in this work support a collision-induced mechanism for direct intramolecular conversion of CO_2_ to O_2_. Specifically, with the dynamics evolving on the ground electronic state of neutral CO_2_, we find that O_2_ is formed via delayed fragmentation, where the delay results from atomic rearrangement of the colliding CO_2_ molecules into a strongly bent geometry. This geometry provides access to the O_2_ dissociation product, without visiting other intermediates. Alternative mechanisms were also theoretically investigated, including the possibility of a collision-induced, non-adiabatic transition of the neutral CO_2_ molecules to electronically excited states (Supplementary Fig. 4), as well as collisional dissociation on the anionic CO_2_^−^ surface following double electron transfer from the Au surface. Although these more complicated processes offer intriguing and potentially exploitable alternative avenues to O_2_ formation, they were not necessary for explaining the experimental observables and were calculated to be less likely under the current experimental conditions.

The mechanism reported here is distinct from previously proposed mechanisms for CO_2_ → C + O_2_ conversion. Specifically, the mechanism differs from that of photochemical interconversion^14^ not only in terms of activation (collisional vs. photochemical) but also because the collisional mechanism occurs via a delayed fragmentation of a single CO_2_ intermediate, i.e., without visiting the linear COO state. The collisional mechanism also differs fundamentally from that taking place in electron-attachment experiments^17^, where the CO_2_ bends spontaneously on the anionic potential energy surface. Instead, the bent CO_2_ state is accessed on the neutral surface via collisional energy transfer. Furthermore, while the collisional interconversion of CO_2_ to O_2_ has comparable efficiency to activation via high-energy photons and higher efficiency than via electron attachment, it is a much simpler process. Importantly, our mechanism is independent of surface temperature and generic to surface composition (tested on Au, Pt, SiO_2_, In_2_O_3_, SnO_2_) as long as: (i) the surface contains atoms heavier than the constituents of CO_2_ and (ii) surface charging is mitigated when CO_2_ ions are used. Finally, we note that an analogous dissociation reaction: OCS → C + SO, previously reported^27^ for OCS^+^ collisions on Ag(111), was speculated to occur via a sharply bent excited state, such as the OCS(^3^*A*), activated either by neutralization prior to impact or by the energetic collision with the surface. However, the basic mechanistic features of the latter process—including whether it involves unimolecular collisions or Eley–Rideal reactions with surfaced-absorbed O or S atoms—were not addressed.

The intramolecular CO_2_ reaction may be relevant in astrochemical environments with abundant CO_2_ and prevalent solar wind. Solar ultraviolet light photo-ionizes CO_2_ molecules readily, producing ions which are then picked up by the solar wind and accelerated to hyperthermal energies^28,29^. Collisions of these fast ions with the surfaces of dust particles or other astrophysical bodies can activate the dissociation. Such interactions may affect dynamically the composition of cometary comae, contributing to the abundance of the super-volatiles O_2_ and CO. Production of O_2_ from CO_2_ was explicitly disregarded in the coma of comet 67P early on (pre-perihelion) during the Rosetta mission, owing to the low abundance of CO_2_ and poor correlation between O_2_ and CO_2_ fluxes^6^. However, the situation may warrant reexamination in the post-perihelion phase, when CO_2_ can reach abundancies as high as 32% relative to H_2_O, a 10-fold increase versus pre-perihelion^30^. The precise level of contribution to the O_2_ abundance in the coma cannot be determined without CO_2_ ion energy and flux data. Nevertheless, the number is likely small for collisional encounters on dust and cometary surfaces. Even at low yield, however, contribution to the measured O_2_ abundance may be disproportionate if the CO_2_ reaction occurs close to the point of measurement. For example, we have verified experimentally that the reaction takes place on indium–tin oxide (ITO), a man-made material found on Rosetta’s thermal insulation and solar panels. Thus, CO_2_ collisions on the spacecraft’s exposed surfaces can change the composition of the surrounding gaseous halo with unknown repercussions for mass spectrometric measurements^31^.

Similar collisional processes may have occurred in early Earth when projectiles, such as meteorites, traversed through its CO_2_-dominated atmosphere; likewise, orbiting satellites/spacecraft or high-speed space debris^32^ will encounter neutral or photoionized CO_2_ in Mars’ upper ionosphere. In these situations, the target surface is moving against a stagnant CO_2_ atmosphere with correspondingly high velocities, driving the partial transformation of CO_2_ into O_2_. Indeed, O_2_ abundances in the 1000’s of parts per million measured at Mars^33^ may contain significant contributions from such processes.

Finally, although the yield of O_2_ is relatively small in the current study, a combination of collisional activation with photoexcitation, electron attachment, and Eley–Rideal reactions in a plasma reactor may result in a process that could be promising for CO_2_ reduction strategies, as well as plasma-driven continuous O_2_ production in CO_2_ atmospheres.

## Methods

### Experimental

All experiments were carried out in a custom-made low-energy ion scattering apparatus^34^. The CO_2_^+^ ion beam was extracted from an inductively coupled plasma, struck in a reactor held at 2 mTorr using a CO_2_/Ar/Ne gas mixture supplied with 500 W RF power at 13.56 MHz. Ions were delivered to a grounded surface at 45° incidence angle; typical beam currents of 5–15 μA were spread over a ~3 mm spot. Beam energy was varied between 40 and 200 eV by externally adjusting the plasma potential. The beam energy distribution had a Gaussian shape with a FWHM of ~5 eV. Typical target surfaces were polycrystalline Au foils (5 N), sputter-cleaned with an Ar^+^ ion gun before each run. Scattered ion products, exiting at an angle of 45° in the scattering plane, were energy-resolved and mass-resolved using an electrostatic ion energy analyzer and a quadruple mass spectrometer, respectively. All ions were detected using a channel electron multiplier, biased as appropriate to detect positive or negative ions. Differences in detector bias precluded a direct comparison of signal intensities between product ions of different charge polarities. All collected signals were normalized to the beam current measured on the sample.

### Simulations

MD trajectories of CO_2_ scattering from Au(111) surface were propagated on a potential energy surface represented as a sum of the CO_2_ electronic energy, the molecule–surface interaction energy, and the interatomic potential of the metal atoms. The CO_2_ energy of the ground singlet electronic state was modeled by a self-consistent-charge tight-binding model, the molecule–surface interaction energy was described by a Tersoff–Brenner reactive force field, and the interatomic metal interactions were represented by an embedded-atom potential. Both the tight-binding model and reactive force field were specifically parameterized for the CO_2_–Au system on datasets consisting, respectively, of 689 and 930 ab initio energies with mean absolute errors of 0.42 and 0.48 eV, which are small compared to the translational and internal energies involved in the collisions. Finite-temperature tight-binding calculations were used to alleviate concerns about the multi-reference nature of the dissociating scattering products. Non-adiabatic transition probabilities presented in Supplementary Fig. 4 were computed by integration of the time-dependent Schrödinger equation, together with on-the-fly multi-reference complete active space self-consistent field calculations of the participating ground and excited electronic states. The Langevin equation with a friction coefficient that described the dissipation of energy in electron–hole pair excitations in the metal was integrated to propagate the motion of the atoms. The Au(111) surface was modeled by a (8 × 8 × 6) slab of Au atoms with 2D periodic boundary conditions imposed in the *x* and *y* directions. Trajectories were initiated with fixed incidence angle of 45° and with center-of-mass of the CO_2_ molecules at 5.5 Å above an energy-minimized gold surface, and they were terminated after the scattering products had left the surface region. The trajectories were averaged over molecular orientation and surface unit cell, and 0.2 eV thermal ro-vibrational energy was attributed to the internal degrees of freedom of CO_2_. 20,000 trajectories were computed for each incidence energy and all scattering products were collected for final analysis. The probability for ion formation depended exponentially on the inverse of the normal component of the center-of-mass velocity and the coupling to the metal, and the exit velocities of the ions were corrected for the ionization energies.

### Competing mechanisms

Before considering an intramolecular reaction, two competing processes must be excluded: (1) sputtering of O_2_ from the surface and (2) abstraction of atomic O from the surface to form the hypothetical radical •CO_3_ via an Eley–Rideal reaction, followed up by spontaneous dissociation to CO + O_2_. Sputtering can be discounted for two reasons: (a) sputtering requires typically higher *E*_0_ to induce the collision cascade—indeed, there is some evidence of sputtering in the O_2_^+^ energy distributions for *E*_0_ > 100 eV, forming a low-energy shoulder to the main peak (Fig. 1b) and (b) the sputtering peak position varies little with *E*_0_—indeed, the main O_2_^+^ peak separates from the low-energy shoulder entirely for *E*_0_ > 180 eV (Fig. 1b). Moreover, there is no evidence of sputtering in the O_2_^−^ energy distributions (Fig. 1c). Similarly, formation of O_2_ by Eley–Rideal reactions likely does not occur, owing to low sticking probability for O on Au. In addition, abstraction reactions slow down the exiting product moiety and would result in O_2_^±^ exit energies much lower than those measured^20^. Isotopic scattering experiments with C^16^O_2_^+^ on ^18^O-covered Pt surfaces have confirmed that an Eley–Rideal reaction is possible and that it yields slower ^16^O^18^O^+^ than the simultaneously observed ^16^O^16^O^+^ products (Supplementary Figs. 5 and 6). The energy peaks from CO_2_^+^ scattering on Au show no such contribution at lower energies—the peaks are much narrower. Therefore, neither O_2_ sputtering nor O-atom abstraction by CO_2_ on Au surfaces occurs to a degree that would affect the conclusions of our study.

## Supplementary information

Supplementary Information

Peer Review File

Description of Additional Supplementary Files

Supplementary Video 1

## Data Availability

All relevant raw data, experimental and computational, are available from the authors upon request.

## Code availability

The computational code is available from the authors upon request.
