# Publisher Correction: Direct dioxygen evolution in collisions of carbon dioxide with surfaces

**DOI:** 10.1038/s41467-019-10728-6

**Published:** 2019-06-18

**Authors:** Yunxi Yao, Philip Shushkov, Thomas F. Miller, Konstantinos P. Giapis

**Affiliations:** Division of Chemistry and Chemical Engineering, California Institute of Technology, Pasadena, CA 91125 USA

**Keywords:** Chemical physics

Correction to: *Nature Communications* https://www.nature.com/articles/s41467-019-10342-6, published online 24 May 2019.

The original version of this Article contained errors in Fig. 1. The titles at the top of each panel a, b, c were incorrectly given as ‘CO_2_^+^/Au → CO_2_^+^ A’, ‘CO_2_^+^/Au → CO_2_^+^ B’ and ‘CO_2_^+^/Au → O_2_^–^ C’ instead of the correct panel a, b, c ‘CO_2_^+^/Au → CO_2_^+^’, ‘CO_2_^+^/Au → O_2_^+^’ and ‘CO_2_^+^/Au → O_2_^–^’, respectively. This has been corrected in the PDF and HTML versions of the Article.

