# Supplementary material for: Direct dioxygen evolution in collisions of carbon dioxide with surfaces
Source: Nat Commun. 2019 May 24;10:2294. doi: 10.1038/s41467-019-10342-6 (PMC6534623; doi:10.1038/s41467-019-10342-6)
Supplement: Supplementary file 1 — Supplementary Information [file 41467_2019_10342_MOESM1_ESM.docx]

Supplementary Figures

for

**Direct dioxygen evolution in collisions of carbon dioxide with surfaces**

Yunxi Yao, Philip Shushkov, Thomas F. Miller III, and Konstantinos P. Giapis

Division of Chemistry and Chemical Engineering, California Institute of Technology, Pasadena, California 91125, USA

*Email: [giapis@cheme.caltech.edu](mailto:giapis@cheme.caltech.edu)


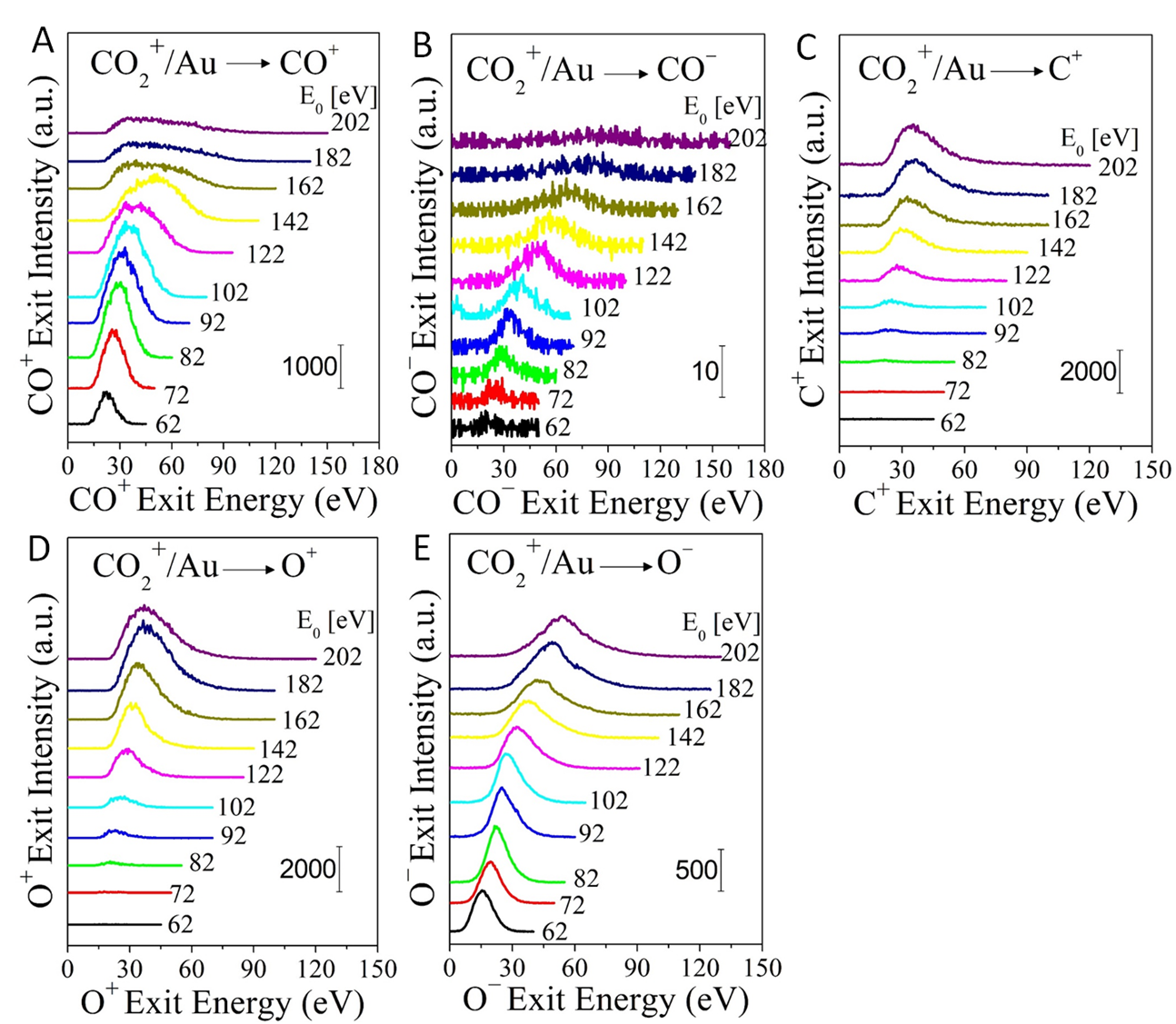
Supplementary Figure 1. **Fragmentation products in CO_2_^+^ collisions with Au surfaces**. Product energy distributions of (A) CO^+^, (B) O^+^, (C) C^+^, (D) O^+^, and (E) O^−^ ion exits from CO_2_^+^/Au for various CO_2_^+^ incidence energies (E_0_) as annotated on each panel. Signal intensities between corresponding positive and negative ion exits cannot be compared due to differences in detector bias.

Supplementary Figure 2. **Comparison of experimental vs. calculated O_2_¯ formation selectivity as a function of CO_2_^+^ incidence energy.** The selectivity is computed as the ratio I(O_2_¯)/[I(O_2_¯)+I(O¯)]. The error bars represent one standard deviation across 10 samples of 2000 trajectories each from the ensemble of molecular dynamics trajectories.

Supplementary Figure 3. **Calculated exit velocity distributions of scattering products from CO_2_ collisions with Au surfaces at 56.4 eV incidence energy.** Full lines depict the exit velocity distributions of the ions and dashed lines depict those of the parent neutral products. “Partial” refers to O atoms produced via partial dissociation of CO_2_, and “full” refers to O atoms produced via full dissociation. The overlap of the O^–^ distributions formed via partial and full dissociation determines the wide breath of the summed O^–^ distribution. The shifts in the relative positions of the neutral scattering products result from the energy requirements of the different dissociation channels, whereas the shifts between the distributions of the ions and their parent neutrals result from the energy needed for ionization and the exponential dependence of the ionization probability on the inverse of the normal velocity component with respect to the surface.

Supplementary Figure 4. **Collision-induced non-adiabatic transitions in CO_2_ scattering on Au surfaces.** (A) Electronic energy of the ground (blue) and lowest few excited states of A’ (full lines) and A” (dashed lines) symmetry of a CO_2_ molecule along a representative scattering trajectory. As the CO_2_ collides with the surface, the molecule bends and reaches a conical intersection (CI) region, where the ground and first A’ excited state become close in energy. (B) Averaging over an ensemble of 2000 trajectories of initially ro-vibrationally hot CO_2_ molecules shows that nearly 70% of the scattering trajectories reach the CI region (red). From the trajectories that have reached the CI region about 40% undergo a non-adiabatic transition to the first excited state (blue), which brings the total collision-induced non-adiabatic flux to 28%.


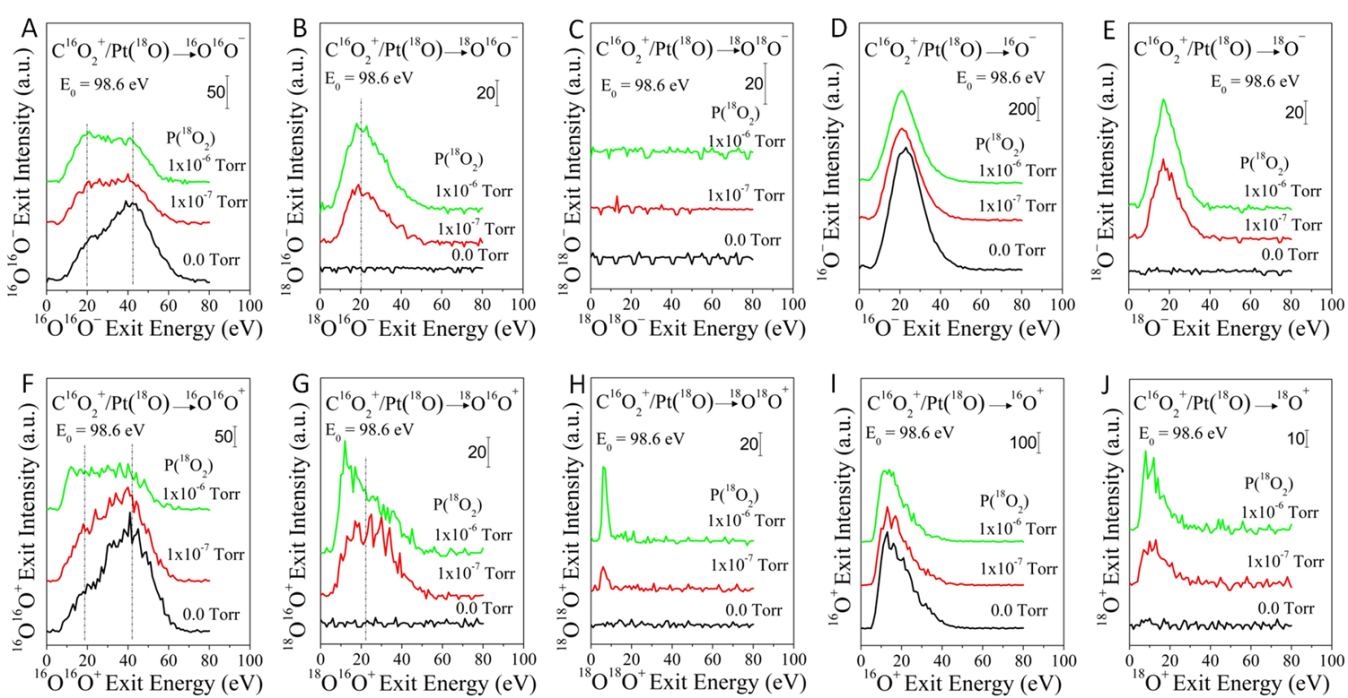


Supplementary Figure 5. **CO_2_^+^ collisions with ^18^O-atom covered Pt surfaces as a function of coverage.** Product energy distributions of (A) ^16^O^16^O^−^, (B) ^18^O^16^O^−^, (C) ^18^O^18^O^−^, (D) ^16^O^−^, (E) ^18^O^−^ ,(F) ^16^O^16^O^+^, (G) ^18^O^16^O^+^, (H) ^18^O^18^O^+^,(I) ^16^O^−^ , and (J) ^18^O^+^ ion exits from CO_2_^+^/Pt(^18^O) for CO_2_^+^ with an incidence energy of E_0_=98.6 eV at various ^18^O_2_ exposure pressures as annotated on each panel. When the Pt surface is covered with ^18^O, an Eley-Rideal reaction product ^18^O^16^O can be formed between surface ^18^O and ^16^O from within the incident CO_2_^+^. The ^18^O^16^O product is indeed observed in both charge polarities but it exits at low energy (~20 eV) as expected. In contrast, the ^16^O^16^O product of the intramolecular reaction exits at high energy (~42 eV) in both charge polarities.


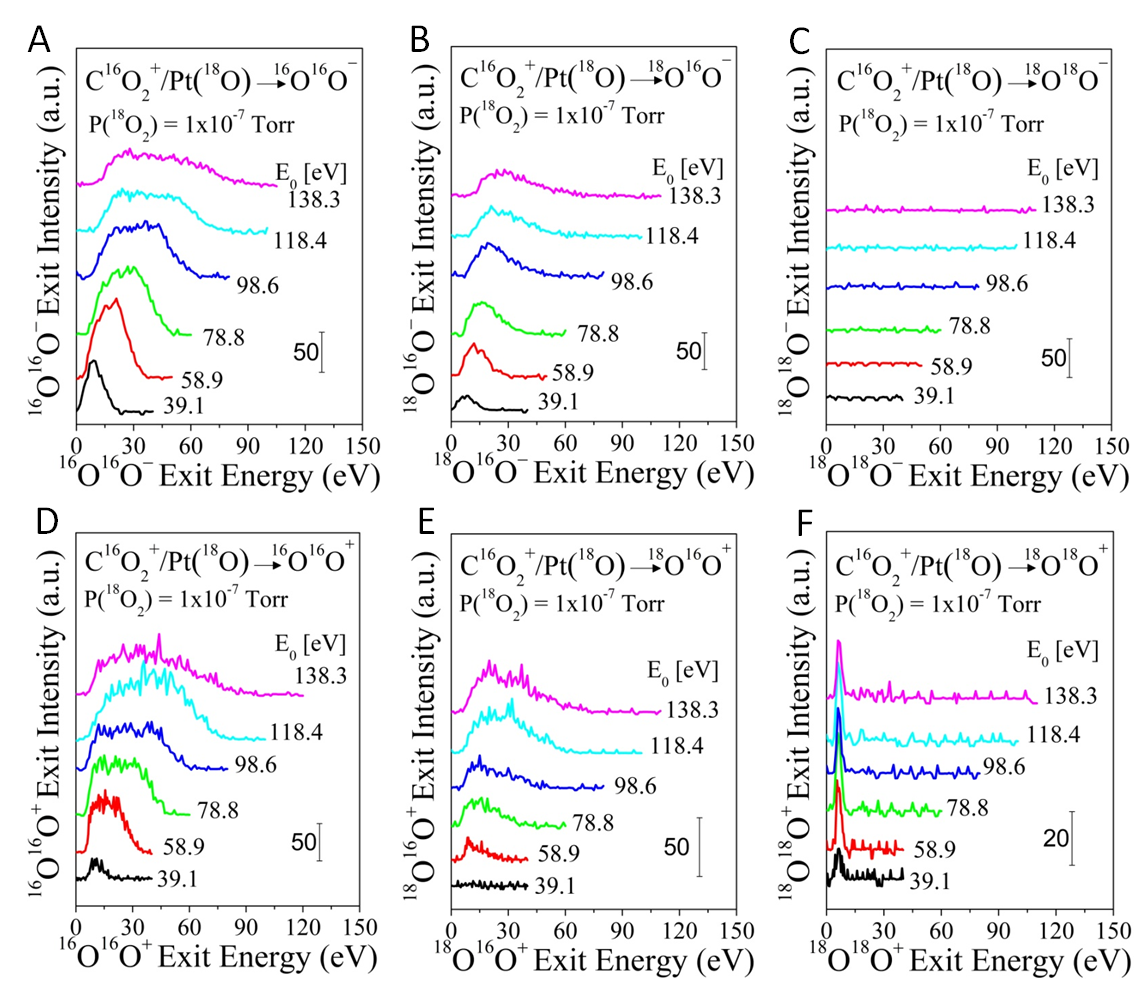


Supplementary Figure S6. **CO_2_^+^ collisions with ^18^O-atom covered Pt surfaces as a function of incidence energy.** Product energy distributions of (A) ^16^O^16^O^−^, (B) ^18^O^16^O^−^, (C) ^18^O^18^O^−^, (D) ^16^O^16^O^+^, (E) ^18^O^16^O^+^, and (F) ^18^O^18^O^+^ ion exits from CO_2_^+^/Pt(^18^O) at various incidence energies as annotated on each panel. When the Pt surface is covered with ^18^O, the Eley-Rideal reaction product ^18^O^16^O, formed between surface ^18^O-atoms and ^16^O from within the incident CO_2_^+^, exits the surface with low kinetic energy (Panels B & E). In contrast, the energy distributions of the intramolecular reaction product ^16^O^16^O are bimodal, extending to higher exit energies (Panels A & D). The high exit energy part of the ^16^O^16^O^±^ distributions is inconsistent with an origin in Eley-Rideal reactions or surface sputtering.
