# Supplementary material for: Direct dioxygen evolution in collisions of carbon dioxide with surfaces
Source: Nat Commun. 2019 May 24;10:2294. doi: 10.1038/s41467-019-10342-6 (PMC6534623; doi:10.1038/s41467-019-10342-6)
Supplement: Supplementary file 3 — Description of Additional Supplementary Files [file 41467_2019_10342_MOESM3_ESM.docx]

**Description of Supplementary Files**

**File Name:** Supplementary Video 1

**Description:** Visualization of the complete CO2 collision trajectory discussed in Figure 3B of the main text. The trajectory illustrates the formation of O2 via bending of the CO2 molecule during scattering from the gold surface.
